# Membrane-associated human tyrosinase is an enzymatically active monomeric glycoprotein

**DOI:** 10.1371/journal.pone.0198247

**Published:** 2018-06-05

**Authors:** Nicole J. Kus, Monika B. Dolinska, Kenneth L. Young, Emilios K. Dimitriadis, Paul T. Wingfield, Yuri V. Sergeev

**Affiliations:** 1 Ophthalmic Genetics and Visual Function Branch, National Eye Institute, National Institutes of Health, Bethesda, Maryland, United States of America; 2 Trans-NIH Shared Resource on Biomedical Engineering and Physical Science, National Institute of Biomedical Imaging and Bioengineering, National Institutes of Health, Bethesda, Maryland, United States of America; 3 Protein Expression Laboratory, National Institute of Arthritis and Musculoskeletal and Skin Diseases, National Institutes of Health, Bethesda, Maryland, United States of America; Wageningen Universiteit, NETHERLANDS

## Abstract

Human tyrosinase (hTyr) is a Type 1 membrane bound glycoenzyme that catalyzes the initial and rate-limiting steps of melanin production in the melanosome. Mutations in the *Tyr* gene are linked to oculocutaneous albinism type 1 (OCA1), an autosomal recessive disorder. Currently, the application of enzyme replacement therapy for a treatment of OCA1 is hampered by the absence of pure hTyr. Here, full-length hTyr (residues 1–529) was overexpressed in *Trichoplusia ni* larvae infected with a baculovirus, solubilized with detergent and purified using chromatography. Michaelis-Menten kinetics, enzymatic specific activity, and analytical ultracentrifugation were used to compare the hTyr in detergent with the soluble recombinant intra-melanosomal domain, hTyrC_tr_ (residues 19–469). Active hTyr is monomeric in detergent micelles suggesting no stable interactions between protein molecules. Both, hTyr and hTyrC_tr_, exhibited similar enzymatic activity and ligand affinity in L-DOPA and L-Tyrosine reactions. In addition, expression in larvae is a scalable process that will allow high yield protein production. Thus, larval production of enzymatically active human tyrosinase potentially could be a useful tool in developing a cure for OCA1.

## Introduction

Pigmentation, specifically eumelanin and pheomelanin synthesis, is a process restricted to melanocytes and the retinal pigment epithelium (RPE) [1]. While there are several genes involved in pigmentation, the *Tyr* gene, responsible for the production of the tyrosinase protein (EC 1.14.18.1), is the most important. Human tyrosinase, hTyr, a type 1 glycoenzyme, located in the membrane of melanosomes, is the key enzyme involved in pigment production. Mutations in hTyr result in pigmentation disorders, causing over- or under-expression of tyrosinase linked with either hyper- or hypopigmentation, respectively. A lack or decreased activity of tyrosinase causes a disorder known as oculocutaneous albinism type 1 (OCA1), which affects the skin, hair, and eyes of individuals. This hypopigmentation is also characterized by distinctive ocular changes [2]. OCA1 is further divided into two subtypes, OCA1A and OCA1B, depending on the severity of the mutation.

In OCA1A, the mutation in *Tyr* is severe and produces an incomplete or inactive tyrosinase enzyme. Such mutations cause the protein to be degraded and retained in the endoplasmic reticulum (ER) by the quality control mechanism (ERQC), leading to a lack of melanin production [3, 4]. Individuals diagnosed with OCA1A have typical disease phenotypes [2]. Subsequently, these individuals experience nystagmus and poor visual acuity throughout their life as their RPE fails to develop melanin.

In OCA1B, the mutation in *Tyr* ranges in severity and often produces a partially active enzyme. These mutations cause a marked decrease in the expression of melanogenic enzymes. Individuals affected with OCA1B have characteristic white or off-white hair, eyebrows, and lashes, all of which may darken throughout their life as melanin develops [2]. Consequently, their irises may remain blue or develop different color later in life. While nystagmus persists, it can dampen in speed and amplitude as these individuals age.

The hTyr polypeptide is composed of 529 amino acids predicting a polypeptide molecular weight of approximately 67 kDa. Several structural elements found in the human tyrosinase include a signal peptide sequence at the N-terminus (residues 1–18), an intra-melanosomal domain (residues 60–476), and a C-terminus transmembrane domain involved in the anchoring of the protein (residues 474–496) [5, 6].

Human tyrosinase is a complex protein and to be properly folded and expressed, it must undergo post-translational modification, including heavy glycosylation [6]. Only seven asparagine (N) residues have been determined to be glycosylated with an N-x-S/T conserved sequence motif. These N-glycan motifs are conserved amongs tyrosinases from mouse and human and have been shown to be involved in maturation, stability, function, and protein sorting [7].

Biochemically, hTyr catalyzes the initial and rate-limiting steps of melanogenesis and is assisted by two proteins: tyrosinase-related-protein 1 (Tyrp1/gp75) and dopachrome tautomerase (Tyrp2/DCT). Both of these participate in the pathway of melanin biosynthesis [8]. In the pathway, tyrosinase hydroxylates L-tyrosine to L-dihydroxyphenylalanine (L-DOPA), which is subsequently oxidized to transient L-dopaquinone before dopachrome is formed. hTyr contains two metal binding sites in its active site, CuA and CuB, which bind copper atoms with six coordinating histidine residues H180, H202, H211, H390, H363, and H367 [9]. These coppers form a peroxo bridge with oxygen, driving the hydroxylation of L-tyrosine [10]. As hTyr matures, it is folded in the ER before it is passed through the Golgi. However, hTyr is not fully functional until it reaches the melanosome membrane [11]. The intracellular transport of hTyr is highly controlled and regulated by copper uptake, as well as by the N-glycosylation process [12].

The soluble intra-melanosomal domain of tyrosinase (residues 19–469) was expressed and biochemically characterized previously [5]. Consequently, we used the soluble intra-melanosomal domain of tyrosinase to create albinism-related mutants, which enabled us to correlate tyrosinase enzymatic activity and mutant variant protein stability [13]. Here we express, purify, and characterize full-length hTyr. Moreover, we have shown that hTyr solubilized in detergent micelles has a weight-average molecular weight of 57 kDa, indicating that it exists as a monomeric protein. Both hTyr and monomeric hTyrC_tr_ exhibit similar activities, enzymatic turnover, and ligand affinity. In addition, expression in larvae is a scalable process that will allow high yield protein production. Thus, larval production of enzymatically active human tyrosinase potentially could be a useful tool in developing a cure for OCA1.

## Results and discussion

Recombinant hTyr was expressed and produced in whole *Trichoplucia ni* (*T*. *ni*) larvae. Due to insolubility of full length tyrosinase, the protein was purified in the presence of detergents. We utilized both Triton X-100 and its reduced optically transparent variant, hydrogenated Triton X-100 (hTriton X-100), to successfully purify hTyr. Since Triton X-100 has an intrinsically high UV absorbance and fluorescence hTriton X-100 was critical for confirming spectrophotometric monitoring without increased UV interference.

**Fig 1:**
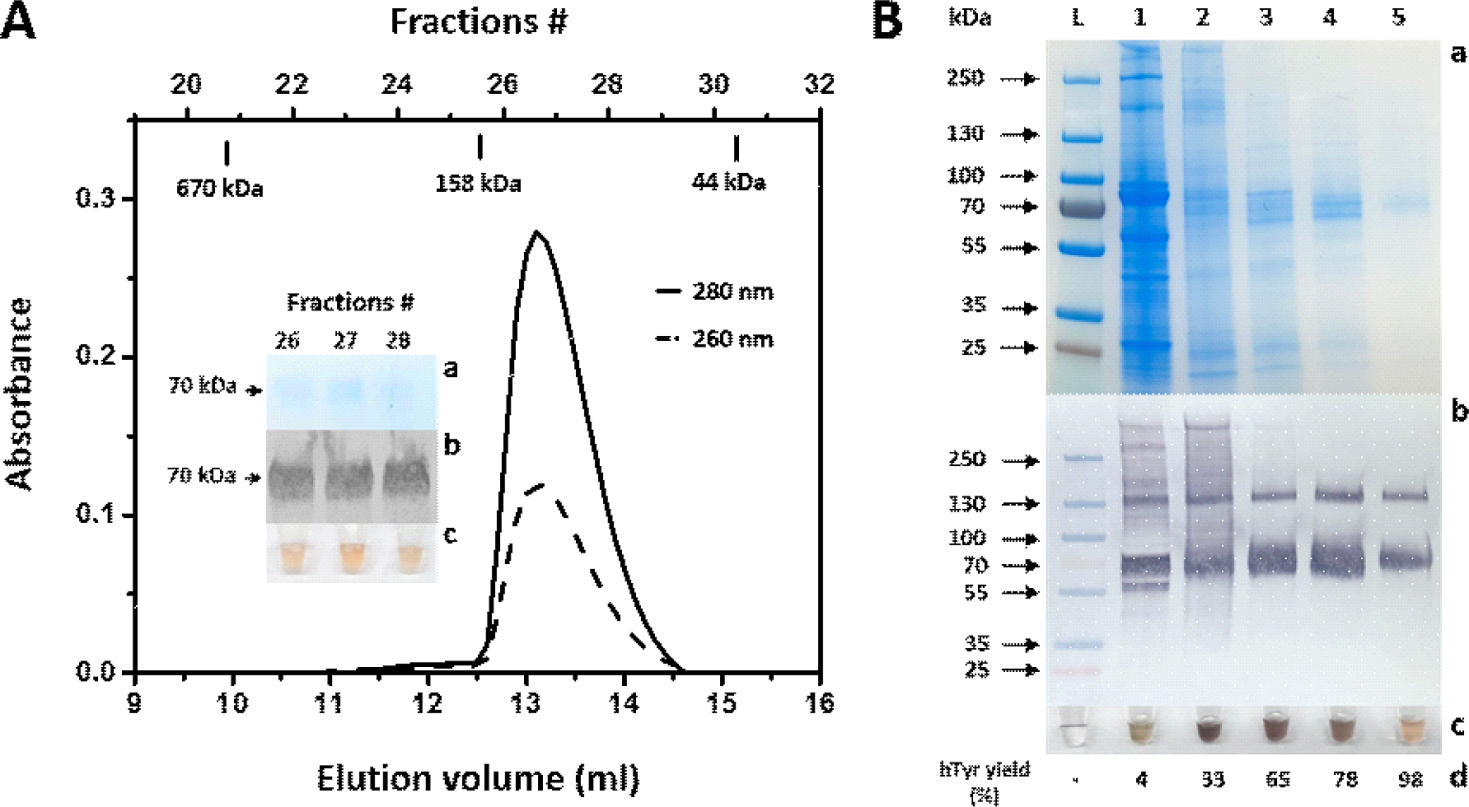
Recombinant hTyr purified from *T*.*ni*. larval biomass in presence of hydrogenated Triton X-100. **Panel A**: hTyr eluted from Superdex 200 increase 10/300 GL column after Ni-NTA gravity purification. The absorption was measured at 280 nm (black solid line) and 260 nm (black dashed line). The following Bio-Rad SEC standards are shown at the top of the panel: Thyroglobulin (670 kDa), γ-globulin (158 kDa), and ovalbumin (44 kDa). The insert shows SDS-PAGE (**a**), Western blot (**b**), and diphenol oxidase activity (**c**) for the corresponding fractions containing hTyr. Arrows display the protein ladder marker at 70 kDa. **Panel B:** SDS-PAGE (**a**) and Western blot (**b**) shows stepwise purification of hTyr. From the left: L, protein ladder; 1, total lysate of larvae expressing hTyr; 2, sample after 5 ml HisTrap crude column; 3, sample after Sephacryl S-300 16/60 HR SEC; 4, sample after Superdex 200 increase 10/300 GL SEC; 5, sample after Ni-NTA affinity chromatography. For the western blot anti-tyrosinase antibody (T311, 1:2000, Santa Cruz Biotechnology) was used. (**c**) shows the corresponding diphenol oxidase activity of hTyr measured after 30 min of incubation at 37°C with 3 mM L-DOPA in 50 mM sodium phosphate buffer, pH 7.4. **(d)** reveals the hTyr yields in protein extracts during purification, which were obtained from the SDS-PAGE gels using UN-SCAN-IT gel ^TM^ gel analysis software (Silk Scientific, Inc.).

Using hTriton X-100, hTyr was, thus, purified by immobilized-metal affinity and size-exclusion chromatography (IMAC and SEC, respectively). The purity was monitored by SDS-PAGE and Western blot analyses (Fig 1, S1 Fig). Both analyses showed bands with a molecular mass ~ 70 kDa for recombinant hTyr. The heterogeneity of the protein’s molecular weight can be attributed to the N-linked glycosylation of the enzyme [5]. Using a colorimetric reaction with L-DOPA, which analyzes dopachrome production [14, 15], we determined whether the recombinant hTyr was active. While brown fractions were substantial to indicated activity (Fig 1 B inset c; S1 Fig b insert), orange-brown fractions exemplified tyrosinase activity, as well as a greater quantity of the protein of interest.

**Fig 2:**
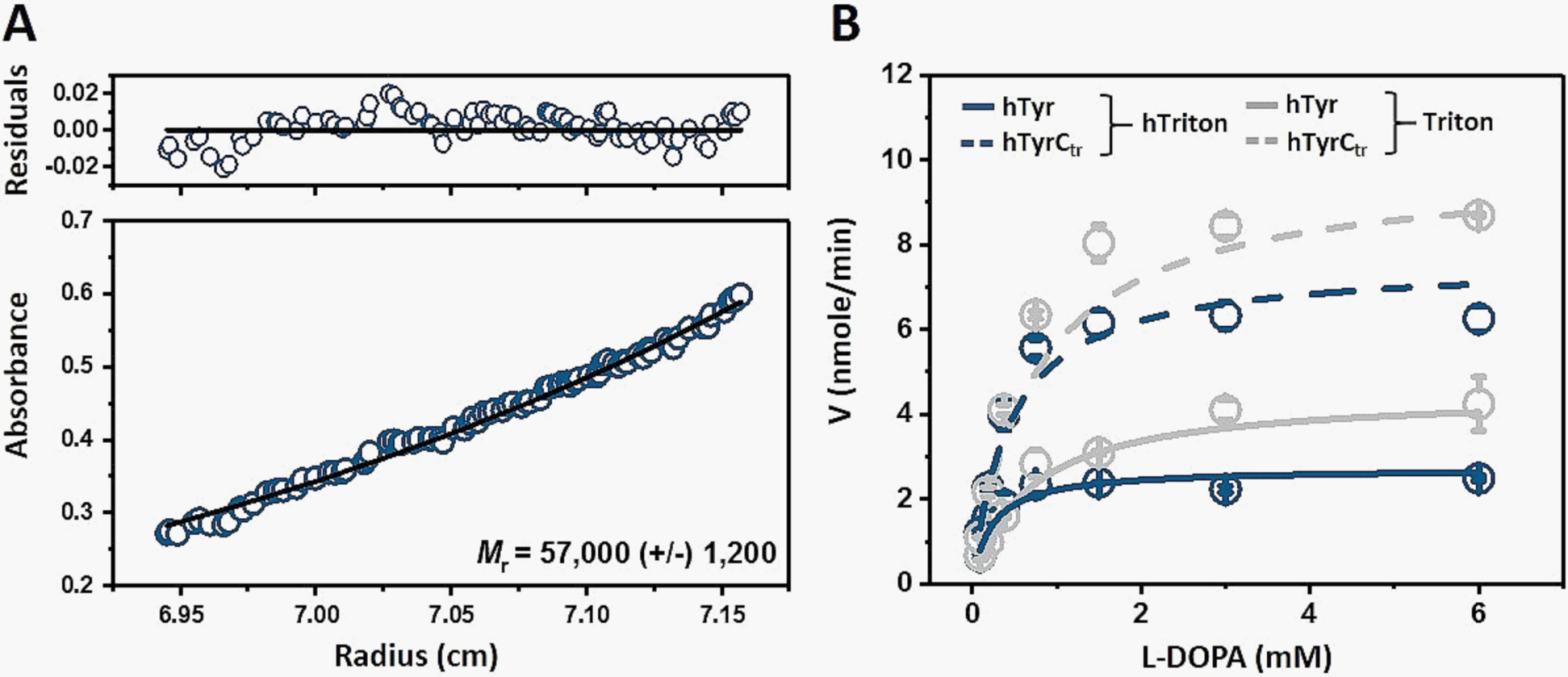
Sedimentation equilibrium and diphenol oxidase activity of the recombinant hTyr. **Panel A** shows sedimentation equilibrium of hTyr in 0.1% hTriton X-100. The protein concentration gradients at 280 nm are shown after 16 h at 15,000 rpm. In the bottom panels, the data (open circles) were best fitted in a single ideal species (continuous lines). The difference in fit between the model and data are shown as residuals in the top panel; the random distribution indicates a good fit. **Panel B** shows Michaelis-Menten plots of diphenol oxidase activity of hTyr (solid line) and hTyrC_tr_ (dashed line) in the presence of 0.1% hTriton X-100 (blue) or 0.1% Triton X-100 (grey) as a functions of L-DOPA concentration measured at 37°C. The lines represent nonlinear fits to the Michaelis-Menten equation obtained from OriginPro software. Error bars represent the standard deviations.

SEC and analytical ultracentrifugation (AUC) were then used to determine the oligomeric state of hTyr. SEC indicated a molecular weight of ~141 kDa in hTriton X-100 (Fig 1A). This result was contrasted by sedimentation equilibrium, where the effect of the detergent was balanced during a sample preparation (see Methods) and the weight-average molecular weight was determined to be ~57 kDa in hTriton X-100 (Fig 2A). The western blots had heterogeneous bands, which were attributed to the protein’s glycosylation, as demonstrated by the diffuse bands on the blots and an observation of tyrosinase polypeptides with varying MWs (Fig 1B). Therefore, the centrifugation result for hTyr was obtained assuming a 10% carbohydrate content to maintain the best fit for the sedimentation equilibrium curve.

The enzymatic activity of hTyr solubilized in detergent was tested *in vitro* with L-DOPA, which is known to interact with mammalian tyrosinases (http://brenda-enzymes.info). The Michaelis-Menten constant (K_m_) and maximal velocity (V_max_) of hTyr were calculated from the Michaelis-Menten plot in the presence of Triton X-100 and hTriton X-100 (Fig 2B, Table 1). For hTyr in the presence of hTriton X-100, the K_m_, V_max_, and k_cat_, were calculated to be 0.23 ± 0.06 mM, 2.72 ± 0.10 nmol/min, and 1.4 ± 0.1 min^-1^, respectively, while for hTyrC_tr_, they were 0.45 ± 0.06 mM, 7.59 ± 0.60 nmol/min, and 3.8 ± 0.3 min^-1^, respectively. Table 1 also shows corresponding parameters obtained in the presence of Triton X-100. The similarity of the K_m_ values in the presence of hTyr or hTyrC_tr_ shows similar binding affinities for both proteins. The monophenolase activity of tyrosinase was tested in the presence of Triton X-100 (S3 Fig). Values of V_max_ and K_m_ were 0.07 ± 0.01 nmole/min and 0.09 ± 0.03 mM, respectively. Michaelis constants from diphenol oxidase and monophenolase reactions agree with K_m_ values obtained from various literature sources [5,9,10,13,15,29–32] presented in the S1 Table.

**Table 1:** Michaelis-Menten kinetics parameters comparing the purified full-length protein (hTyr) to the purified truncated protein (hTyrC_tr_).

	K_m_ (mM)	V_max_ x 10^−3^ (μmol/min)	k_cat_ (min^-1^)
**hTyr**_**hTriton X-100**_	0.23 ± 0.06	2.72 ± 0.10	13.6 ± 1.0
**hTyrC**_**tr hTriton X-100**_	0.45 ± 0.06	7.59 ± 0.60	38.0 ± 3.0
**hTyr**_**Triton X-100**_	0.67 ± 0.03	4.50 ± 0.12	22.5 ± 1.0
**hTyrC**_**tr Triton X-100**_	0.74 ± 0.04	9.83 ± 0.33	49.1 ± 2.0

Michaelis-Menten kinetics measured in the presence of a detergent, Triton X-100 or hTriton X-100. The k_m_ defines the affinity of L-DOPA for the hTyr and hTyrC_tr_ enzymes. The V_max_ is the maximal rate at which L-DOPA can be converted to dopachrome once bound to the enzymes. k_cat_ is the enzymes turnover, which is the number of substrate molecules turned over per enzyme per minute (Vmax/E_t_; where E_t_ is the concentration of the enzyme (2 μM), which was estimated using the BCA assay).

In the presence of Triton X-100, the specific activity of hTyr was 0.24 ± 0.01 units/mg and for hTyrC_tr_ it was 0.43 ± 0.02 units/mg. Some of the differences in the activities and kinetic parameters of the two proteins might be attributed to an interaction between the transmembrane helix of hTyr and the detergent, suggesting that the removal of the helix does not decrease enzymatic activity of hTyrC_tr_. Thus, the presence of the transmembrane helix at the C-terminus is not responsible for the tyrosinase’s activity.

**Fig 3:**
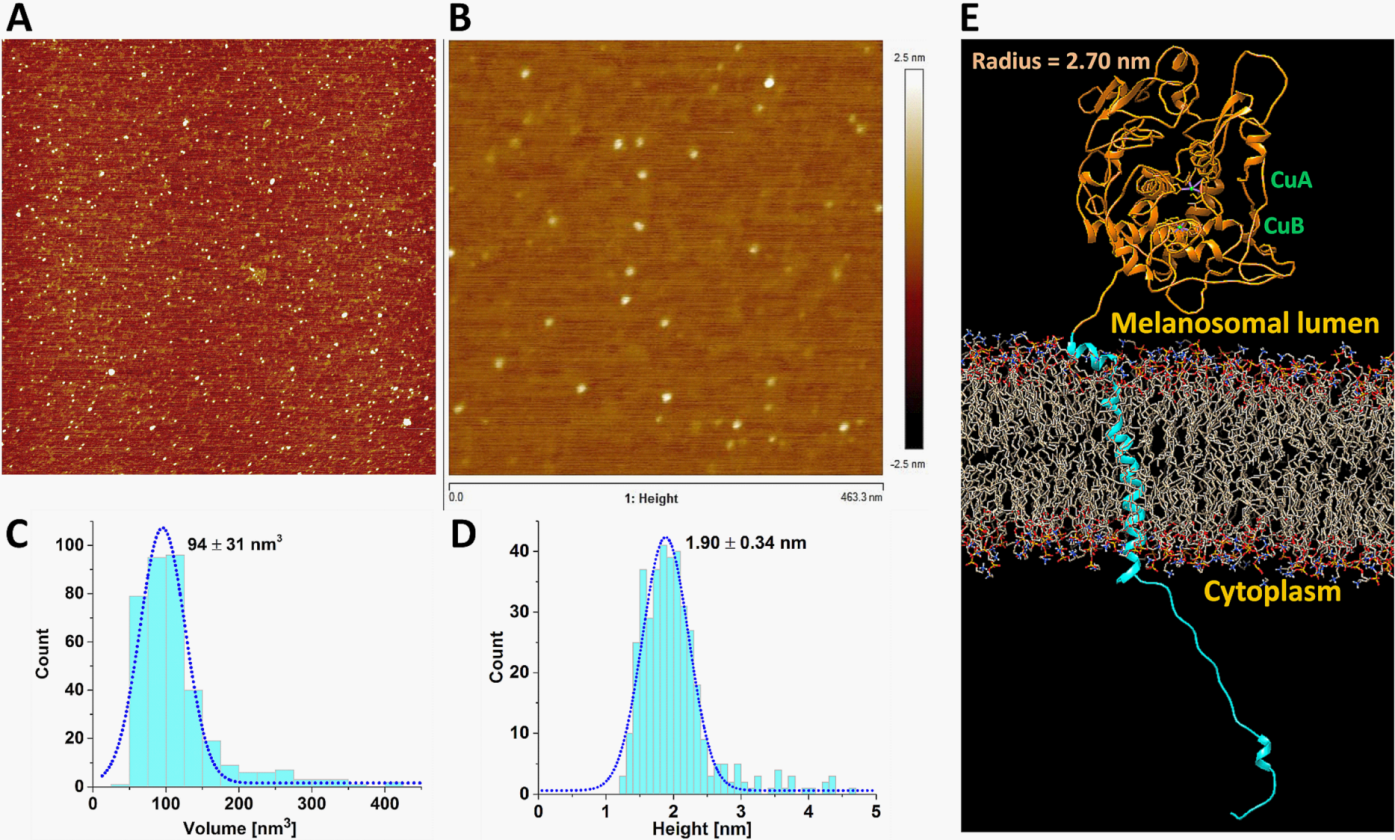
Atomic force microscopy of human tyrosinase intra-melanosomal domain and homology model of full-length human tyrosinase. **Panel A:** a full field image of the protein sample shows a uniform distribution of the protein on the APS mica. **Panel B:** an enlarged fragment of the same image. **Panel C:** particle volume distribution. **Panel D**: particle height distribution showing the molecular size uniformity. **Panel E:** the homology model of human tyrosinase. The globular intra-melanosomal domain and C-terminal domain, which is incorporated in the lipid membrane, and the trans-membrane helix are shown by orange and cyan ribbon structure, respectively. The copper binding sites are shown in green.

To study tyrosinase structure and oligomerization state, atomic force microscopy (AFM) was used (Fig 3**).** Due to the presence of detergent in the membrane protein sample, the intra-melanosomal domain of hTyr [5] was used for AFM analysis. A full field image and the corresponding enlarged image are shown in Fig 3A and 3B, respectively. A distribution of volume measurements shown at Fig 3C indicates that most of the particles from the full field image are protein monomers. The volume is 94 ± 31 nm^3^ (N = 383), which is consistent with the expected volume of ~100 nm^3^ for a typical protein of ~57 kDa.

The monomers have a footprint that is equivalent to a circle of 7–9 nm in the diameter. The particles were uniform in size and their height distribution is shown in the Fig 3D. The particle height was 1.9 ± 0.34 nm (N = 383), with a small population of particles that measured taller than 2.5 nm. From the expected volume of the particle, the equivalent radius of the particle could be evaluated as 2.82 nm. A radius of >2.70 nm was estimated from equivalent volumes of a homology model of the globular intra-melanosomal domain of hTyr, which is shown as the orange ribbon structure in Fig 3E. The domain shows no propensity to oligomerize in native conditions.

In this work, we showed that both, hTyr and hTyrC_tr_, exhibited similar activity, enzymatic turnover, and ligand affinity in an enzymatic reaction (Table 1), which agrees with previous descriptions of the tyrosinase enzyme [5, 16, 17]. From the SEC, hTyr exhibited a molecular weight larger than 100 kDa (Fig 1, S2C Fig), which can be attributed to the protein eluting as a protein–detergent complex. Both Triton X-100 and hTriton X-100 micelles had molecular weight of ~ 90 kDa. Therefore, a monomeric protein in either Triton X-100 or hTriton X-100 would be expected to run as a ~ 150 kDa complex if a one micelle to protein ratio is assumed. In contrast, a weight-average molecular weight of 57 kDa, obtained using sedimentation equilibrium in hTriton X-100, shows that the purified tyrosinase is a monomeric glycoprotein, excluding the possibility for tyrosinase to form a dimer in the presence of the detergent.

The molecular weight estimates could be related to lower SEC accuracy, varying amounts of carbohydrates, and/or the number of detergent molecules bound to the protein molecule when forming a micelle. Our results illustrate that in the presence of detergent, tyrosinase forms a monomer. This also agrees with results of previous studies on the intra-melanosomal domain of tyrosinase [5, 13] and our current AFM results (Fig 3), suggesting a lack of stable interaction between the soluble tyrosinase domains. However, we cannot exclude the possibility of a tyrosinase oligomerization in a lipid-rich environment where tyrosinase is anchored to the melanosome membrane. We might expect that in the membrane, the local protein concentration could be very high, driving self-association to form dimers or even higher oligomers. In addition, as previously been noted by Yurkow and Laskin [18], in tumor homogenates of B16/C3 melanoma from mice, tyrosinase exists in a number of discrete isozymes with molecular weights ranging from 58–150 kDa.

Through our results of purification, we believe that the tyrosinase intra-melanosomal domain plays a key role in maintaining the molecule catalytic activity. This is further validated in Fig 2E, which exemplifies a fragment of approximately 60 residues removed from the C-terminus of tyrosinase to engineer the hTyrC_tr_, which has the close enzymatic activity as previously stated.

Fully active tyrosinase glycoenzyme could potentially be used for a treatment of OCA1 ocular disorder. In some cases, enzyme replacement therapy can help compensate for the enzyme shortage due to abnormal enzyme function or loss-of-function genetic mutations. There are several successful instances of the enzyme replacement therapy treatments for lysosomal defects, such as Fabry disease, which is a rare X-linked disorder caused by deficient activity of the lysosomal enzyme α-galactosidase A, Pompe disease; and others [16]. In Fabry disease the recombinant enzyme was used for reversing the pathogenesis of the chief clinical manifestations of this disease [19]. In future, liposomal delivery of active tyrosinase to melanosomes might alleviate the effects of mutated tyrosinase caused by genetic mutations. In addition, expression in larvae is a scalable process that will allow high yield protein production [5, 12, 13, 20]. Although post-translational modifications in hTyr expressed in the larval system indicated changes to glycans related to the baculovirus system [5], we believe that the larval production of enzymatically active human tyrosinase potentially be a useful tool in the treatment of OCA1.

In conclusion, we have described methods that can be used to express, purify, and characterize the full-length hTyr. Producing pure hTyr is important in not only the characterization of its enzymatic abilities, but also in drug targeting studies and eventually the synthesis of artificial human liposomes containing a full length tyrosinase that can be used in the treatment of OCA-1. Thus, larval production of enzymatically active human tyrosinase potentially potentially is a useful tool in a cure for OCA1.

## Materials and methods

### Protein expression and purification

Protein expression and purification was performed as described in accompanying protocol [21]. Briefly, recombinant full-length human tyrosinase was commercially cloned, expressed, and produced in whole *T*. *ni* larvae (Allotropic Tech, MD), as previously described for the recombinant hTyrC_tr_ [5]. A 6xHis-Tag was added to the C-terminus to facilitate protein purification as previously shown [21]. The synthetic genes were cloned into the baculovirus vector and co-transfected into Sf9 cells. The virus was then injected into the *T*. *ni* larvae and larval biomass was produced and frozen at -80^°^C. The frozen biomass was homogenized and solubilized using detergents.

The homogenate of the larval biomass was prepared in the lysate buffer with 1.0% Triton X-100 or hTriton X-100) and sonicated for 10 minutes continuously using an Ultrasonic Processor GE130PB (Hielscher System, Germany). hTyr was purified by IMAC, and hTyr was eluted with a gradient of 0–500 mM imidazole including either 0.1% Triton X-100 or 0.1% hTriton X-100. Fractions, which contained hTyr, were identified by their color change in presence of L-DOPA and then dialyzed, concentrated, and further purified by SEC. Columns were calibrated using gel filtration standards (BioRad, CA): thyroglobulin (670 kDa), ү-globulin (158 kDa), ovalbumin (44 kDa), myoglobin (17 kDa), and vitamin B12 (1.4 kDa).

The presence of tyrosinase in fractions was confirmed by SDS-PAGE and Western blotting using the anti-tyrosinase T311 monoclonal mouse antibody (Sigma-Aldrich, CA).

### Protein concentration measurement

hTyr concentration in presence of hTriton X-100 was measured spectrscopically at 280 nm using a NanoDrop 2000 (Thermo Scientific). However, Triton X-100 interferes with the UV absorption and and prevents the measurement of protein concentration by using spectral methods. Therefore, the bicinchoinic acid (BCA) assay was used to measure the total protein concentration of hTyr according to standard protocols (Thermo Fisher Scientific, MD).

### Tyrosinase enzymatic assays

Enzyme diphenolase activity was determined using L-DOPA as a substrate (Sigma Aldrich, MO) as in a previously described absorption assay [22, 23]. The reaction was performed in a 96-well plate. Each well contained a mixture of 3.0 mM L-DOPA and the 0.1 mg/mL protein concentrate in Triton X-100, pH 7.4. The reaction was incubated for 30 min at 37^°^C and was monitored at 475 nm for dopachrome formation (Ɛ_dopachrome_ = 3700 M^-1^ cm^-1^) on a SpectraMax i3 multimode detection platform (Molecular Devices, CA).

### Kinetic parameters

The monophenolase and diphenol oxidase reaction rates (*V*) of hTyr and hTyrC_tr_ were determined using L-tyrosine and L-DOPA as substrates in concentration ranging from 0.023 to 0.75 mM and 0.094 to 6 mM, respectively. Enzymatic assays were performed in 10 mM sodium phosphate buffer, at pH 7.4 in the presence of either Triton X-100 or hTriton X-100. The reactions were incubated at 37°C and monitored at 475 nm for dopachrome formation with a SpectraMax i3 Multi-Mode Detection Platform (Molecular Devices, CA). The Michaelis-Menten constant (K_m_) and maximal velocity (V_max_) of the proteins were calculated from the Michaelis-Menten plots, which were independently fitted with the corresponding nonlinear function using the OriginPro 2015 program (OriginLab Inc., MA).

### Analytical ultracentrifugation

AUC was performed as previously described [24]. A Beckman Optima XL-I analytical centrifuge, absorption optics, an An-60 Ti rotor, and standard double-sector centerpiece cells were used. Sedimentation equilibrium measurements were made at 20°C and concentration profiles were recorded at 260 nm after 16 h at 15,000 rpm. Baselines were established by over-speeding at 45,000 rpm for 3 h. Data (the average of eight scans collected using a radial step size of 0.001 cm) were analyzed using the standard Optima XL-I data analysis software. Protein partial specific volume was calculated from the amino acid composition, and solvent densities were estimated using the program SEDNTERP (http://www.rasmb.bbri.org/). To estimate of the partial specific volume of the protein–hTriton X-100 complex (as detergent binding was not directly determined), it was assumed that 1 micelle of detergent bound to one molecule of protein. In this calculation, the micellar size of hTriton X-100 was assumed to be 95 kDa and that its partial specific volume was 0.91 mL/g. Additionally, a carbohydrate content of ~ 10% was accounted for by using a value of 0.63 mL/g for sugar content. Analytical ultracentrifugation in hTriton X-100 buffer was performed in 54% D_2_0 to balance the effect of the detergent on mass determinations [25].

### Atomic force microscopy

AFM images were acquired in ambient air, on a Multimode-Picoforce AFM instrument (Bruker Nano, Inc., Santa Barbara, CA) using silicon probes (OTESPA, Bruker Nano, Inc.) with nominal tip radius of 7 nm and resonance frequency of ~300 kHz. The images were acquired using the “tapping” or oscillating mode of the instrument whereby the cantilever is vibrated near its resonance. Freshly cleaved mica disk substrates (12 mm) were modified to render them hydrophobic and positively charged by incubating them with aminopropyl-silatrane (APS) solution (0.17 mM) for 30 minutes, rinsing with ultrapure water and drying as described previously [26]. Five microliters of appropriately diluted solutions of highly purified human tyrosinase hTyC_tr_ were deposited onto the APS-mica and incubated for 15 minutes, then gently rinsed with ultrapure water to remove salt, and dried in a nitrogen stream before imaging. The modified mica substrates ensured that the strongly negatively charged proteins would firmly attach to allow imaging. Images were pre-processed with the instrument software (Nanoscope Analysis, v 8.15) and then analyzed using the particle analysis features of NIH Image (ImageJ) software.

### Homology modeling

The amino acid sequence of hTyr (isoform 1) was retrieved from the UniProt database (Reference #P14679; http://www.uniprot.org/uniprot). The atomic structures for the tyrosinase intra-melanosomal domain and full-length protein were modeled using the molecular visualization, modeling, and dynamics program, YASARA (www.yasara.com). Crystal structures of the human tyrosinase related protein 1 [6], tyrosinase from *Bacillus megaterium* [27], and oxy-form of the copper-bound *Streptomyces castaneoglobisporus* tyrosinase complexed with a caddie protein [28] were used as structural templates. In total, in the full length human tyrosinase structure was modeled for amino acid residues from 19 to 525. The structure of the protein was incorporated into a lipid membrane consisting of phosphatidyl-ethanolamine molecules, and the protein-membrane complex was optimized and equilibrated using 250 picosecond simulated annealing in water.

## Supporting information

S1 Table Michaelis-Menten constant (K_m_) of human tyrosinases from different sources. (DOCX)

S1 Fig Purification steps of hTyr in the presence of hydrogenated Triton X-100. **Panel A**: IMAC using a GE Healthcare HisTrap crude 5 mL column. The dashed line indicates the imidazole gradient up to 100%. **Panel B**: SEC performed with a Sephacryl S-300 16/60 HR column. **Panel C:** SEC of hTyr with a Superdex 200 increase 10/300 GL column. The gray shadow in each panel shows the fractions containing hTyr. The inserts show Western blots (a) and L-DOPA activity assays (b). Arrows display the protein ladder marker at 70 kDa.
(TIF)

S2 Fig Purification steps of hTyr in the presence of Triton X-100. **Panel A**: IMAC using a GE Healthcare HisTrap crude 5 mL column. The dashed line indicates the imidazole gradient up to 100%. **Panel B**: SEC was performed with a Sephacryl S-200 16/60 HR column. **Panel C:** SEC of hTyr (black line) with a Superose 12 10/300 GL column. The red line shows the Bio-Rad SEC standards: Thyroglobulin (670 kDa), γ-globulin (158 kDa), ovalbumin (44 kDa), and myoglobulin (17 kDa). The fractions containing hTyr. The inserts show Western blots (a) and L-DOPA activity assays (b). b). Arrows display the protein ladder marker at 70 kDa.
(TIF)

S3 Fig Kinetic analysis of hTyr. Michaelis-Menten plot of the monophenolase activity of hTyr, as a function of L-tyrosine concentrations. The enzyme assay was conducted at 37°C in the presence of 0.1% Triton X-100. The red line represents the nonlinear fit to the Michaelis-Menten equation obtained from the OriginPro software. The experiment was performed in duplicate and error bars represent standard deviations.
(TIF)
