# Supplementary material for: Membrane-associated human tyrosinase is an enzymatically active monomeric glycoprotein
Source: PLoS One. 2018 Jun 5;13(6):e0198247. doi: 10.1371/journal.pone.0198247 (PMC5988326; doi:10.1371/journal.pone.0198247)
Supplement: S1 Table — (DOCX) [file pone.0198247.s001.docx]

| **Source** | **K_m_ (mM)** | | **comments** | **literature** |
| --- | --- | --- | --- | --- |
|  | **L-tyrosine** | **L-DOPA** |  |  |
| ***E. coli*** | 0.17 | 0.36 |  | [29] |
| ***E. coli*** | - | 0.31 |  | [30] |
| **Sf9 cell lines** | 0.22 | 0.34 | MBTH^2^ | [10] |
| **HEK293-TYR** | - | 0.48 |  | [31] |
| **TXM13 pigment cells** | - | 0.49 |  | [31] |
| **melanoma** | - | 0.4 |  | [32] |
| **RVH 421 human malignant melanoma cells** | 0.1 ^*^ | 0.7 ^**^ | ^*14^CO_2_ assay  ^**^MBTH^2^ | [15, 31] |
| **Cos7 cells** | 0.079 | 0.46 | MBTH^2^ | [9] |
| ***T. ni* larvae^1^** | 0.16 | 0.46 | Intramelanosomal domain^3^ | [5] |
| ***T. ni* larvae^1^** | - | 0.85 | Intramelanosomal domain^4^ | [13] |
| ***T. ni* larvae^1^** | 0.09 | 0.23  0.67 | hTriton X-100^5^  Triton X-100 | Present work |
| ***T. ni* larvae^1^** | - | 0.45  0.74 | hTriton X-100^5^  Triton X-100  Intramelanosomal domain^4^ | Present work |

**S1 Table.** Michaelis*-*Menten constant (K_m_) of human tyrosinases from different sources.

^1^Cabbage looper (*Trichoplusia ni*) larvae

## ^2^MBTH, 3-Methyl-2-benzothiazolinone hydrazine

^3^dopachrome absorption measured at 490 nm

^4^dopachrome absorption measured at 475 nm

^5^hydrogenated Triton X-100
