# Supplementary figures and images for: Membrane-associated human tyrosinase is an enzymatically active monomeric glycoprotein
Source: PLoS One. 2018 Jun 5;13(6):e0198247. doi: 10.1371/journal.pone.0198247 (PMC5988326; doi:10.1371/journal.pone.0198247)

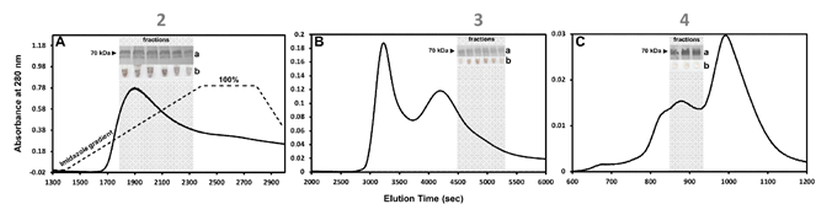

Supplement: S1 Fig — Panel A: IMAC using a GE Healthcare HisTrap crude 5 mL column. The dashed line indicates the imidazole gradient up to 100%. Panel B: SEC performed with a Sephacryl S-300 16/60 HR column. Panel C: SEC of hTyr with a Superdex 200 increase 10/300 GL column. The gray shadow in each panel shows the fractions containing hTyr. The inserts show Western blots (a) and L-DOPA activity assays (b). Arrows display the protein ladder marker at 70 kDa. (TIF) [file pone.0198247.s002.tif]

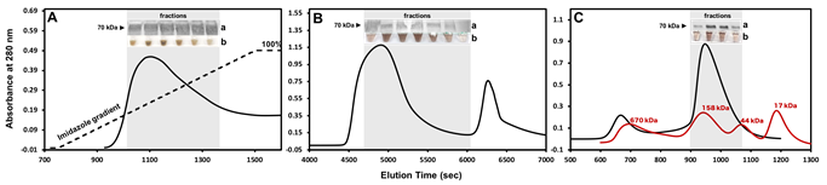

Supplement: S2 Fig — Panel A: IMAC using a GE Healthcare HisTrap crude 5 mL column. The dashed line indicates the imidazole gradient up to 100%. Panel B: SEC was performed with a Sephacryl S-200 16/60 HR column. Panel C: SEC of hTyr (black line) with a Superose 12 10/300 GL column. The red line shows the Bio-Rad SEC standards: Thyroglobulin (670 kDa), γ-globulin (158 kDa), ovalbumin (44 kDa), and myoglobulin (17 kDa). The fractions containing hTyr. The inserts show Western blots (a) and L-DOPA activity assays (b). b). Arrows display the protein ladder marker at 70 kDa. (TIF) [file pone.0198247.s003.tif]

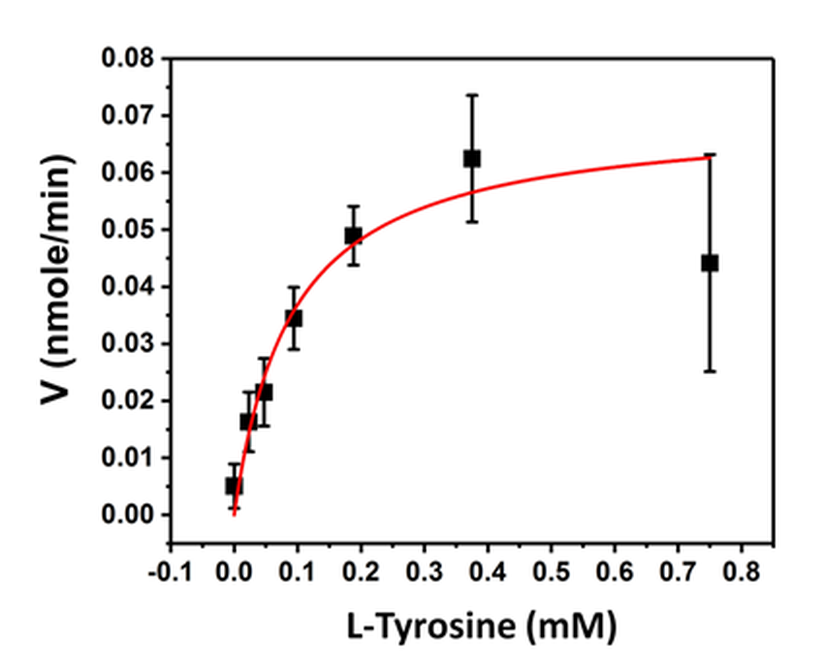

Supplement: S3 Fig — Michaelis-Menten plot of the monophenolase activity of hTyr, as a function of L-tyrosine concentrations. The enzyme assay was conducted at 37°C in the presence of 0.1% Triton X-100. The red line represents the nonlinear fit to the Michaelis-Menten equation obtained from the OriginPro software. The experiment was performed in duplicate and error bars represent standard deviations. (TIF) [file pone.0198247.s004.tif]
